# Tail Risk Spillover Between Global Stock Markets Based on Effective Rényi Transfer Entropy and Wavelet Analysis

**DOI:** 10.3390/e27050523

**Published:** 2025-05-14

**Authors:** Jingjing Jia

**Affiliations:** Department of Accounting, Business School, Beijing Language and Culture University, Beijing 100083, China; jiajingjing@blcu.edu.cn

**Keywords:** global stock markets, tail risk, information spillover, effective Rényi transfer entropy, wavelet analysis

## Abstract

To examine the spillover of tail-risk information across global stock markets, we select nine major stock markets for the period spanning from June 2014 to May 2024 as the sample data. First, we employ effective Rényi transfer entropy to measure the tail-risk information spillover. Second, we construct a Diebold–Yilmaz connectedness table to explore the overall characteristics of tail-risk information spillover across the global stock markets. Third, we integrate wavelet analysis with effective Rényi transfer entropy to assess the multi-scale characteristics of the information spillover. Our findings lead to several key conclusions: (1) US and European stock markets are the primary sources of tail-risk information spillover, while Asian stock markets predominantly act as net information receivers; (2) the intensity of tail-risk information spillover is most pronounced between markets at the medium-high trading frequency, and as trading frequency decreases, information spillover becomes more complex; (3) across all trading frequencies, the US stock market emerges as the most influential, while the Japanese stock market is the most vulnerable. China’s stock market, in contrast, demonstrates relative independence.

## 1. Introduction

The integration of global financial markets has become a prominent topic in recent research. This integration occurs across traditional financial markets, such as equity, bond, foreign exchange markets, and the banking system [1,2,3], as well as between traditional and modern financial markets. Wątorek et al. demonstrated a significant correlation between cryptocurrency markets and traditional financial markets [4]. The growing interconnectedness increases the vulnerability of the global financial system. As a result, shocks can be quickly transmitted among global markets. In the context of increasing financial market integration, numerous empirical studies have investigated how major global events contribute to cross-market risk spillovers, such as the COVID-19 pandemic [5,6,7] and the Russia-Ukraine conflict [8,9]. Although many of these studies adopt event-based approaches, our study instead employs tail-risk as a proxy for major global shocks and uses a wider range of time series to study the characteristics of risk spillover. Moreover, recent research often focuses on regional markets. For example, some studies examine emerging markets such as the ASEAN-4 [10,11], the BRICS [12,13], and other developing economies [14,15]. However, given the reality of global financial integration, understanding the impact of extreme events requires including both developed and emerging markets. Therefore, our study adopts a broader perspective by incorporating a diverse set of markets.

Tail risk refers to the risk associated with extreme values [16] and is therefore widely used in measuring extreme events in financial markets [17,18,19], commodity markets [20,21,22], and other fields. Various methodologies have been proposed to measure tail-risk spillovers, such as CoVaR and Delta CoVaR [23,24], Granger causality in risk [25,26], and GARCH family models [7,27,28,29]. However, these approaches overlook the interconnectedness within the system. Unlike traditional methods, the financial network approach, which integrates econometric methods with network topology theory, has received significant attention. Härdle et al. [30] and Fan et al. [31] proposed the TENET (Tail-Event driven NETworks) framework. Xu et al. analyzed the tail-risk interdependence among 23 cryptocurrencies and identified the systemically important cryptocurrencies using the TENET approach [32]. Similarly, Hu and Guo employed the TENET model to examine tail risk contagion within ESG industries, revealing strong correlations during crises and limited contagion with traditional industries [33]. Additionally, Ando et al. proposed a quantile connectedness approach [34], which has been increasingly applied to study risk spillovers. For instance, Mensi et al. examined the quantile connectedness between eight green bonds and the S&P 500 index, highlighting that green bonds exhibit stronger connectedness with the S&P 500 during crisis, while demonstrating relatively lower volatility during extreme events [35]. Although information-spillover analysis has become a popular tool in risk-spillover research [36], it has rarely been applied to extreme-event transmission. Accordingly, we investigate tail-risk spillovers based on tail-risk information spillover.

Information entropy has proven to be helpful in measuring the complexity of information flow. Transfer entropy, proposed by Schreiber in 2000 [37], is derived from Shannon entropy [38]. Compared with other measures of information entropy, transfer entropy describes the direction in which information flows more explicitly. Due to this characteristic, it has been extensively used to examine information spillovers between stock markets, providing valuable insights into the efficiency and intensity of risk contagion [33]. Kwon and Yang applied transfer entropy to observe the magnitude and directionality of information flows between stock indices, revealing that the United States serves as the primary source of information transmission, while the Asia/Pacific region acts as the predominant recipient [39]. Ji et al. investigated information spillover between global real estate markets by employing an entropy-based network analysis framework for real estate investment trusts (REITs). Their findings identified the strongest pairwise transfer entropy, which is from the United States to Australia [36]. Xie et al. developed a transfer entropy network for global stock indices, revealing a substantial increase in information flows during major financial crises. Their analysis identified stock indices in the Asia-Pacific, Middle East, and Africa as the primary recipients of information, whereas indices in the Americas and Europe emerged as the dominant sources [40]. Different from previous studies, Jizba et al. first applied Rényi transfer entropy to analyze asymmetric information flow between global stock markets [41]. Rényi transfer entropy is distinguished by its ability to precisely measure information spillover within targeted segments of probability distributions [42]. Li et al. demonstrated that Rényi transfer entropy in financial networks can effectively capture extreme market conditions [43]. Since its development, Rényi transfer entropy has been applied in several studies. For example, Dimpfl and Peter employed Rényi transfer entropy to examine the dynamic interactions between US and European stock markets throughout the financial crisis of 2007–2009 [44]. Similarly, Korbel et al. utilized both transfer entropy and Rényi transfer entropy to study information flows among communities of the five largest financial markets, identifying nonlinear interactions during extreme events [42]. These advancements underscore the growing interest in analyzing tail risk contagion through the lens of information entropy. However, the application of Rényi transfer entropy in analyzing tail-risk information spillovers remains relatively limited. To address this gap and enrich the current body of research, this study employs effective Rényi transfer entropy to systematically examine tail-risk information spillovers across global stock markets.

The wavelet approach demonstrates distinct advantages in multi-scale decomposition of time series into both time dimension and frequency dimension [45]. Economic and financial research over the past decade has demonstrated the effectiveness and efficiency of wavelet analysis. The research proves that there are multi-scale features in the stock markets [46,47], the cryptocurrency markets [48], and the interactions between stock markets and commodity markets [49,50,51]. The wavelet method is always combined with the traditional GARCH model to study the multi-scale spillovers between time series. For example, Huang proposed a wavelet-based multi-resolution BEKK-GARCH model to investigate spillover effects across financial markets, highlighting its effectiveness in modeling return and volatility spillovers [52]. Naysary and Shrestha applied a wavelet-based DCC-GARCH model to explore the co-movement between FinTech and ESG markets [53]. Chiranjivi and Sensarma combined the ARMA-GARCH model with wavelet analysis to analyze multi-scale characteristics of time series [54]. However, few studies have examined information spillovers from a frequency perspective. Therefore, we combine information entropy with wavelet analysis for the first time to explore the multi-scale characteristics of tail-risk information spillover across stock markets.

In summary, this paper analyzes tail risk spillover from an information perspective. We first apply effective Rényi transfer entropy to analyze information spillover between stock markets. Then, a wavelet model is used to decompose time series to uncover the multi-scale features of information spillover. Several conclusions are drawn from the study. First, in general, US and European stock markets serve as the primary sources of information spillover related to extreme events, whereas Asian stock markets predominantly function as net receivers of such information. Second, the intensity of information spillover is most pronounced among markets at medium-to-high trading frequencies, and as trading frequency decreases, the spillovers become increasingly complex. Third, across all trading frequencies, the US stock market emerges as the most influential, while the Japanese stock market is the most vulnerable to external information. In contrast, China’s stock market demonstrates relatively greater independence from the influences of other markets.

Several contributions are made by our study. First, we introduce effective Rényi transfer entropy to analyze tail-risk information spillover among global stock markets. This approach provides insights into tail-risk contagion from an information perspective, extending research on extreme event contagion. Second, by employing the framework of Diebold and Yilmaz [55], we examine the distinct roles of global stock markets in information spillovers. The results can help investors make better investment decisions and assist governments in crisis prevention based on their countries’ roles in the global market. Third, we introduce a novel approach by integrating wavelet analysis with effective Rényi transfer entropy, enabling the first comprehensive exploration of multi-scale tail-risk information spillover.

The structure of this paper is as follows: Section 2 outlines the study’s methodology; Section 3 discusses the empirical findings; Section 4 summarizes the conclusions along with relevant policy implications.

## 2. Materials and Methods

### 2.1. Effective Rényi Transfer Entropy

Entropy was first introduced by Clausius in 1865 as a thermodynamic concept to describe the tendency of intensity of heat, pressure, and density to gradually disappear over time. Schwill and Shannon claimed that entropy is applicable to any system with a probabilistic nature [56]. In 1948, Shannon proposed the concept of Shannon entropy, which can be used to quantify the information content of a message [38]. Shannon entropy, $H\left(X\right),$ is defined as follows:

(1) $$H\left(X\right)=-\sum_{x}p\left(x\right)\log_{2}p\left(x\right)$$

where X represents a discrete random variable with probability distribution $p\left(x\right)$; x is the possible outcomes of X.

If two time series X and Y are taken into consideration, the joint entropy of X and Y is applied. It measures the amount of information needed to specify the value of two time series. The joint entropy of X and Y, $H\left(X,Y\right)$, should be defined as:

(2) $$H\left(X,Y\right)=H\left(Y|X\right)+H\left(X\right)=H\left(X|Y\right)+H\left(Y\right)$$

where *H(X)* represents the information only contained in X, while $H\left(Y\right)$ denotes the information solely contained in Y; $H\left(Y|X\right)$ is the conditional entropy of Y given X, and $H\left(X|Y\right)$ is the conditional entropy of X given Y.

Mutual information is another important concept that needs to be clarified. It measures the total amount of information two time series share. It can be defined as:

(3) $$I\left(X;Y\right)=H\left(X\right)-H\left(X|Y\right)=H\left(Y\right)-H\left(Y|X\right)\;=H\left(X\right)+H\left(Y\right)-H\left(X,Y\right)$$

where $I\left(X;Y\right)$ represents the mutual information shared by X and Y.

There are two disadvantages of mutual information. One is, as $I\left(X;Y\right)=I\left(Y;X\right)$, mutual information cannot measure the direction of information flow. The other is mutual information, which includes some part of the information that is statistically shared between time series X and Y. To address this, the conditional mutual entropy between X and Y, given source Z, $I\left(X;Y|Z\right)$, is defined as below.

(4) $$I\left(X;Y|Z\right)=H\left(X|Z\right)-H\left(X|Y,Z\right)$$

where $H\left(X|Z\right)$ is the conditional entropy of X given Z; $H\left(X|Y,Z\right)$ is the conditional entropy of X given both Y and Z.

Assume time series X and Y follow Markov process of degree k and l, respectively. This implies that the state $X_{m+1}$ depends on the k preceding states of X and the l preceding states of Y. Let $X_{m}^{k}$ denote the joint process $\left\{X\left(m\right),X\left(m-1\right),\ldots ,X\left(m-k+1\right)\right\}$, and similarly, let $Y_{m}^{l}$ represent $\left\{Y\left(m\right),Y\left(m-1\right),\ldots ,Y\left(m-l+1\right)\right\}$. By substituting X in Equation (4) with $X_{m+1}$, Y with $Y_{m}^{l}$, and Z with $X_{m}^{k}$, the conditional mutual entropy can be expressed as:

(5) $$I\left(X_{m+1};Y_{m}^{l}|X_{m}^{k}\right)=H\left(X_{m+1}|X_{m}^{k}\right)-H\left(X_{m+1}|Y_{m}^{l},X_{m}^{k}\right)$$

where $I\left(X_{m+1};Y_{m}^{l}|X_{m}^{k}\right)$ measures the conditional mutual entropy of $X_{m+1}$ and $Y_{m}^{l}$ given $X_{m}^{k}$; $H\left(X_{m+1}|X_{m}^{k}\right)$ is the conditional entropy of $X_{m+1}$ given the joint process $X_{m}^{k}$; $H\left(X_{m+1}|Y_{m}^{l},X_{m}^{k}\right)$ represents the conditional entropy of $X_{m+1}$ given both the joint process $X_{m}^{k}$ and the joint process $Y_{m}^{l}$.

The conditional mutual information in Equation (5) is also known as Shannonian transfer entropy (STE) from Y to X. The concept of STE was proposed by Schreiber in 2000 [37]. Unlike mutual information, STE not only captures directional information flows but also identifies dependencies arising from Y. Formally, STE, denoted as $T_{Y\to X}$, can be expressed as:

(6) $$\begin{array}{cc} T_{Y\to X}=I(X_{m+1}; & Y_{m}^{l}X_{m}^{k})\\ & =-\sum p\left(X_{m+1},X_{m}^{k},Y_{m}^{l}\right)log_{2}p\left(X_{m+1}|X_{m}^{k}\right)\\ & +\sum p\left(X_{m+1},X_{m}^{k},Y_{m}^{l}\right)log_{2}p\left(X_{m+1}|X_{m}^{k},Y_{m}^{l}\right)\\ & =\sum p\left(X_{m+1},X_{m}^{k},Y_{m}^{l}\right)log_{2}\frac{p\left(X_{m+1}|X_{m}^{k},Y_{m}^{l}\right)}{p\left(X_{m+1}|X_{m}^{k}\right)} \end{array}$$

where $T_{Y\to X}$ measures the incremental information gained about $X_{m+1}$ when conditioned on both its own historical values and those of Y; $p\left(X_{m+1},X_{m}^{k},Y_{m}^{l}\right)$ is the joint probability distribution of $X_{m+1}$, $X_{m}^{k}$ and $Y_{m}^{l}$; $p\left(X_{m+1}|X_{m}^{k}\right)$ represents the conditional probability distribution of $X_{m+1}$ given $X_{m}^{k}$; $p\left(X_{m+1}|X_{m}^{k}{,Y}_{m}^{l}\right)$ is the probability distribution of $X_{m+1}$ given both $X_{m}^{k}$ and $Y_{m}^{l}$.

While STE is appropriate for estimating transfer entropy when the underlying time series follow a Markov process, real-world data often deviate from such assumptions due to the limitations of finite sample size. As a result, STE estimates may be significantly affected by noise. Marchinski and Kantz proposed the concept of effective transfer entropy (ETE) [57] and applied the surrogate data technique to eliminate the noise. The ETE can be defined as:

(7) $$T_{Y\to X}^{eff}\equiv T_{Y\to X}-T_{Y_{schuffled}\to X}$$

where $T_{Y\to X}$ is the original STE; $T_{Y_{schuffled}\to X}$ represents the transfer entropy computed using the surrogate data technique; $Y_{schuffled}$ denotes the randomized time series that preserves the original series’ mean, variance, and autocorrelation function. Theoretically, all potential correlations between X and $Y_{schuffled}$ are removed, implying that $T_{Y_{schuffled}\to X}$ equals zero. Any non-zero value of $T_{Y_{schuffled}\to X}$ arises from the finite size of the dataset. Equation (7) serves to remove the noise due to the sample size.

Rényi introduced Rényi entropy (RE) in 1970 [58]. Compared to Shannon entropy, Rényi entropy introduces an additional parameter q, which adjusts the weight given to different parts of the probability distribution. RE of order q (q > 0 and q ≠1) of a distribution P on a finite time series X is defined as:

(8) $$S_{q}(P)=\frac{1}{1-q}\log_{2}\sum_{x}p{\left(x\right)}^{q}$$

where X is the time series with probability distribution $p\left(x\right)$; x is the possible outcomes of X.

According to Equation (8), for q > 1, RE exhibits a stronger dependence on the probabilities of high-likelihood events, with this effect becoming increasingly pronounced as q rises. Conversely, for 0 < q < 1, RE emphasizes low-probability events, with greater sensitivity observed as q approaches zero.

Following the same rule of determining the STE, Rényi transfer entropy (RTE) is defined as:

(9) $$T_{q;Y\to X}^{R}=\frac{1}{1-q}\log_{2}\frac{\sum p^{q}\left(X_{m+1}|X_{m}^{k}\right)}{\sum p^{q}\left(X_{m+1}|X_{m}^{k},Y_{m}^{l}\right)}$$

where $T_{q;Y\to X}^{R}$ measures the information flow from Y to X; $\;p^{q}\left(X_{m+1}|X_{m}^{k}\right)\;$ represents the conditional probability distribution of $X_{m+1}$ given $X_{m}^{k}$; $p^{q}\left(X_{m+1}|X_{m}^{k},Y_{m}^{l}\right)$ is the probability distribution of $X_{m+1}$ given both $X_{m}^{k}$ and $Y_{m}^{l}$.

Jizba et al. [41] applied the escort distribution $\varrho_{q}\left(x\right)=\frac{p^{q}(x)}{\sum_{x}p^{q}(x)}$ to normalize the weighted distributions and rewrote RTE as:

(10) $$T_{q;Y\to X}^{R}=\frac{1}{1-q}\log_{2}\frac{\sum \varrho_{q}\left(X_{m}^{k}\right)p^{q}\left(X_{m+1}|X_{m}^{k}\right)}{\sum \varrho_{q}\left(X_{m}^{k},Y_{m}^{l}\right)p^{q}\left(X_{m+1}|X_{m}^{k},Y_{m}^{l}\right)}$$

where $\varrho_{q}\left(X_{m}^{k}\right)$ is the escort distribution of $X_{m}^{k}$; $\varrho_{q}\left(X_{m}^{k},Y_{m}^{l}\right)$ is the joint escort distribution of $X_{m}^{k}$ and $Y_{m}^{l}$.

Rényi transfer entropy (RTE) extends the concept of transfer entropy by incorporating Rényi entropy, enabling analysis of information flow in different parts of the distribution by adjusting the parameter q. When q < 1, RTE emphasizes the tails of the distribution, making it particularly suitable for identifying extreme events and tail dependencies. Unlike STE, RTE can be either positive or negative. A positive value implies that the inclusion of Y’s historical information reduces the tail risk in the predicted distribution of $X_{m+1}$, compared to using X’s history alone. In contrast, a negative value indicates that incorporating the past of both X and Y leads to a fatter tail in the conditional probability distribution of $X_{m+1}$, revealing increased tail risk driven by Y’s influence. Thus, RTE effectively quantifies the incremental tail-risk gain or loss resulting from the inclusion of another market’s historical data. In conclusion, significant RTE under q < 1 serves as a proper indicator of tail-risk spillover.

The effective Rényi transfer entropy is defined as follows:

(11) $$T_{q;Y\to X}^{R,eff}\equiv T_{q;Y\to X}^{R}-T_{q;Yschuffled\to X}^{R}$$

where $T_{q;Y\to X}^{R}$ represents the original Rényi transfer entropy; $T_{q;Yschuffled\to X}^{R}$ denotes the Rényi transfer entropy calculated using the surrogate data method.

### 2.2. Connectedness Framework

Following the connectedness framework proposed by Diebold and Yilmaz [55], we build effective Rényi transfer entropy connectedness tables as shown in Table 1. $R_{ij}$ denotes the pairwise effective Rényi transfer entropy from j to i.

We use effective Rényi transfer entropy (“From” and “To”) to measure the total information spillover between stock markets S. “From” captures the information inflow from other stock markets, and it is computed by the row sum of the pairwise effective Rényi transfer entropy in Table 1. It is defined as:

(12) $$R_{i\to \cdot }=\sum_{j=1}^{N}R_{ij},j\neq i$$

“To” represents the information outflow to other stock markets, computed as the column sum of the pairwise effective Rényi transfer entropy in Table 1. It is defined as:

(13) $$R_{\cdot \leftarrow i}=\sum_{i=1}^{N}R_{ij},i\neq j$$

Net effective Rényi transfer entropy measures a stock market’s net information spillover, derived as the difference between “To” and “From”. It is defined as:

(14) $$R_{i}=R_{\cdot \leftarrow i}-R_{i\to \cdot }$$

The total information spillover of all stock markets is measured by the average of the sum of the “From” or “To”, which is expressed as below:

(15) $$R_{total}=\frac{1}{N}\sum_{i,j=1}^{N}R_{ij},i\neq j$$

### 2.3. Network Construction and Analysis

In the study, we construct tail-risk information spillover networks to capture tail-risk spillover characteristics, using Gephi (0.10.1, Paris, France). According to the complex theory, a network is composed of two elements, nodes and edges. The network can be expressed as:

(16) G=(N,E)

where G is the network; N represents the set of nodes; E is the set of edges.

Let i and j denote nodes in the network. By expanding the edge set E in Equation (16), it can be represented in matrix form:

(17) $$E=\left[\begin{array}{ccc} e_{11} & \ldots & e_{1j}\\ \vdots & \ddots & \vdots \\ e_{i1} & \ldots & e_{ij} \end{array}\right],\;(i,j\in N)$$

where $e_{ij}$ represents the directed edge from i to j; N represents the set of nodes.

In tail-risk information spillover networks, nodes represent a set of stock markets and edges indicate the tail-risk information spillover relationships between stock markets. Edges are directed, and the direction of information flow corresponds to the direction of the edges.

The edge weight, $w_{ij},$ quantifies the magnitude of information transmission from node i to node j, computed using the net pairwise effective Rényi transfer entropy. A higher edge weight indicates stronger tail-risk information spillover between the corresponding stock markets.

The node’s degree equals the total number of its connections. In directed networks, where edges represent directional tail-risk information flow, the degree is typically divided into in-degree and out-degree. The out-degree of a node reflects the number of its outgoing edges. A node with a high out-degree plays a key role in disseminating tail-risk information, exerting broad influence across the network.

The node’s weighted out-degree measures the cumulative strength of its information spillover effects. A greater weighted out-degree signifies a stronger influence over other nodes in the network. It is calculated as:

(18) $$D_{i}^{out}=\sum_{j=1}^{n}w_{ij}$$

where $D_{i}^{out}$ refers to the weighted out-degree of node i; $w_{ij}$ represents the weight of the outgoing edge, $e_{ij}$; n represents the total number of nodes to which node i exhibits spillover effects.

### 2.4. Maximal Overlap Discrete Wavelet Transform

Wavelet transformation is a powerful tool for time series analysis. One of the basic wavelet analysis tools is the Discrete Wavelet Transform (DWT). According to DWT, the decomposition of a time series signal X(t) is determined by two filters, one is the wavelet filter $h_{l}$ and the other one is the scaling filter $g_{l}(l=0,...,L-1)$. The outputs obtained by filtering X(t) with two filters are called the wavelet coefficient and the scaling coefficient. The level j wavelet coefficient $W_{j,t}$ and scaling coefficient $V_{j,t}$ are expressed as follows:

(19) $$W_{j,t}=\sum_{l=0}^{L-1}h_{j,l}X\left(t-l\right)$$

(20) $$\begin{array}{c} V_{j,t}=\sum_{l=0}^{L-1}g_{j,l}X\left(t-l\right) \end{array}$$

Maximal Overlap Discrete Wavelet Transform (MODWT) is proposed by Percival and Walden in 2000 [59] and is a modification of DWT. Similar to DWT, MODWT can perform multi resolution analyses (MRAs) as well. In addition, compared to the DWT, MODWT has several advantages: (1) it can be applied to any sample size, (2) it is non-orthogonal, (3) it offers improved resolution at higher scales, (4) it is translation-invariant, and (5) it provides a more asymptotically efficient wavelet variance estimator. According to the features mentioned above, we employ MODWT in this paper. The wavelet filer ${\tilde{h}}_{j,l}$ and the scaling filter ${\tilde{g}}_{j,l}$ of MODWT are renormalized by DWT filters and defined as below:

(21) $${\tilde{h}}_{j,l}=h_{j,l}/2^{j/2}$$

(22) $${\tilde{g}}_{j,l}=h_{j,l}/2^{j/2}$$

The MODWT wavelet coefficient ${\tilde{W}}_{j}$ and scaling coefficient ${\tilde{V}}_{j}$ at $j^{th}$ level are defined as:

(23) $${\tilde{W}}_{j,t}=\frac{1}{2^{j/2}}\sum_{l=0}^{L-1}h_{j,l}X\left(t-l\right)$$

(24) $$\begin{array}{c} {\tilde{V}}_{j,t}=\frac{1}{2^{j/2}}\sum_{l=0}^{L-1}g_{j,l}X\left(t-l\right) \end{array}$$

The MODWT wavelet and scaling coefficients can also be written in matrix notations as follows:

(25) $${\tilde{W}}_{j}={\tilde{w}}_{j}X$$

(26) $${\tilde{V}}_{j}={\tilde{v}}_{j}X$$

Then, MODWT-based MRA at $j^{th}$ level can be defined as:

(27) $$X=\sum_{j=1}^{J}{\tilde{w}}_{j}^{T}{\tilde{W}}_{j}+{\tilde{v}}_{J}^{T}{\tilde{V}}_{J}=\sum_{j=1}^{J}{\tilde{D}}_{j}+\tilde{S_{J}}$$

where ${\tilde{D}}_{j}$ stands for the $j^{th}$ scale MODWT details of X and $\tilde{S_{J}}$ represents the $j^{th}$ level MODWT smooth of X.

## 3. Results

### 3.1. Data Description

In this study, we selected nine major stock markets in America, Asia, and Europe as our sample. We followed the selection rules of Jizba et al. [41] and made two modifications: (1) we kept only one stock index of the United States, NYSE Composite Index, as NYSE is the largest stock market in the world in terms of market capitalization; (2) we included SSE Composite Index of China and FTSE 100 of UK based on their capitalization rankings among the global stock markets. The basic information on the selected stock markets is summarized in Table 2. Daily closing indices were obtained from Yahoo Finance for the period spanning from 10 June 2014 to 29 May 2024. Given that these markets are located in different time zones, we adopted a methodology similar to that used by Sandoval [60] to address this issue. Specifically, we designated the NYSE as the benchmark. On days when the NYSE was closed but other markets were open, data from those days were excluded. Conversely, if a stock market was closed while the NYSE was open, we substituted its closing price with that from the previous trading day.

To improve time series stationarity, we applied the logarithmic transformation to the closing indices, which is defined as follows:

(28) $$R_{t}=lnP_{t}-lnP_{t-1}$$

where $R_{t}$ is the stock market return at day t; $P_{t}$ is the closing price at day t and $P_{t-1}$ is the closing price at day t − 1.

Table 3 summarizes the descriptive statistics of stock market returns. The return distributions display significant skewness and leptokurtosis, indicating deviations from normality. These findings are further supported by the statistically significant Jarque–Bera test results

Table 4 displays the unconditional correlations among the nine markets’ stock returns, all significant at the 1% level. This highlights global market co-movement, driven by shared information and synchronized responses to shocks. European markets show the strongest correlations, reflecting a higher degree of regional information integration. In terms of cross-regional information dissemination, the correlations between the US stock market and European stock markets are relatively stronger, indicating that the information sharing between the United States and Europe is greater than that between the United States and Asia, as well as between Europe and Asia.

### 3.2. Empirical Results

#### 3.2.1. Overall Analysis of Tail-Risk Information Spillover

As previously noted, when 0 < q < 1, the parameter q of effective Rényi transfer entropy accentuates the influence of tail events, with smaller values of q assigning greater weight to marginal events. However, as q decreases, the results become increasingly sensitive to errors. Consequently, studies typically select a compromise value for q to balance sensitivity and stability [42]. In our study, we chose q = 0.8 for detailed analysis.

We construct the connectedness table of stock markets in Table 5. In Table 5, the row sums, labeled “From”, measure the total effective Rényi transfer entropy received by each of the nine markets from others, capturing tail-risk information spillovers. The results show that the HSI index (0.368) has the highest “From” value, indicating that the Hong Kong market receives the most information from other markets. In contrast, the GDAXI index (0.206) has the lowest value, suggesting that the German market receives the least. Conversely, the column sums, labeled “To”, represent the total effective Rényi transfer entropy transmitted from each market to others, reflecting its contribution to global information flows. The US stock market exhibits the highest total effective Rényi transfer entropy “To” (0.572), whereas the Hong Kong stock market shows the lowest value (0.179). The net effective Rényi transfer entropy, calculated as the difference between the total effective Rényi transfer entropy “To” and “From” is negative for the HSI, N225, SSEC, BSESN, and STI indices. This suggests that all the Asian stock markets primarily act as recipients of information within the global stock market network. In contrast, the US stock market and European stock markets, including the NYA, GDAXI, FTSE, and SSMI indices, display positive net effective Rényi transfer entropy, indicating that these markets are information transmitters and contribute more to the global information flow.

**Figure 1:**
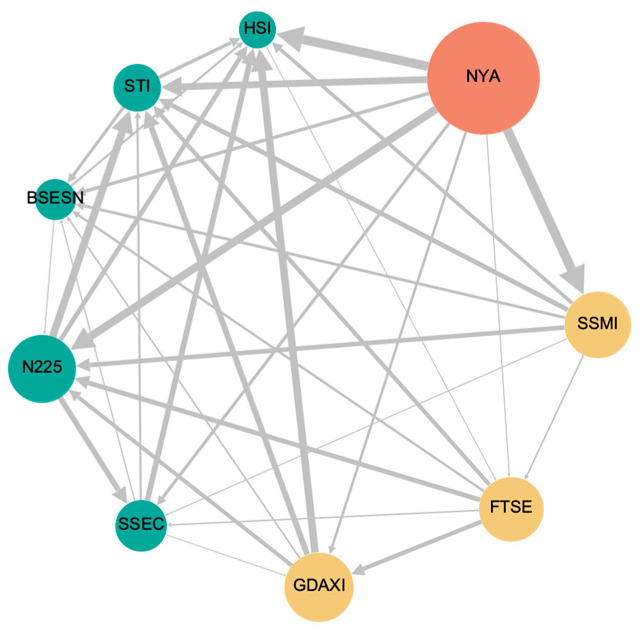
Tail-risk information spillover network based on pairwise net effective Rényi transfer entropy. European markets are represented by yellow nodes, Asian markets by blue nodes, and the US market by an orange node.

We develop a tail-risk information spillover network using pairwise net effective Rényi transfer entropy, as depicted in Figure 1. Nodes in the network are arranged in a clockwise order according to their out-degree. Node size reflects the weighted out-degree, which quantifies a market’s ability to propagate information to others, with larger nodes indicating greater outgoing influence. To emphasize regional characteristics, nodes belonging to the same continent were assigned the same color. The size of edges is proportional to the weight of the edges, signifying the strength of the information spillover.

As illustrated in Figure 1, the US stock market ranks first with the highest out-degree (out-degree = 8), meaning information on extreme events spreads to all other eight markets. This dominance is further emphasized by its largest node size. Additionally, the edges between the NYA, STI, and HSI markets are the thickest, highlighting the US market’s significant role in the global financial network. European markets follow the US market in terms of influence, while Asian markets, with the lowest out-degree, rank last. This suggests that Asian markets have the least influence compared to the US and European markets.

Based on the previous results, it is evident that tail-risk information spillovers exhibit distinct regional characteristics. The US stock market is the most influential, with a substantial amount of tail-risk information originating from the US and affecting other markets, particularly those in Asia. This can largely be attributed to the fact that the US stock market is among the most developed markets, offering significant advantages in various aspects, including market capitalization, liquidity, investor composition, industry structure, and regulatory frameworks [61]. European stock markets also serve as key information transmitters. In contrast, with limited market power, Asian stock markets are primarily receivers of tail-risk information spillovers and are more susceptible to the impact of extreme events compared to other markets. We reach a similar conclusion with Choi and Yoon [62], which underscores that although the importance of Asian markets has grown in recent years, their influence on developed markets still remains relatively constrained.

#### 3.2.2. Time-Frequency Analysis of Tail-Risk Information Spillover

**Figure 2:**
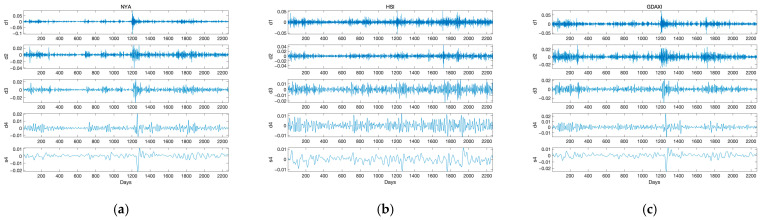

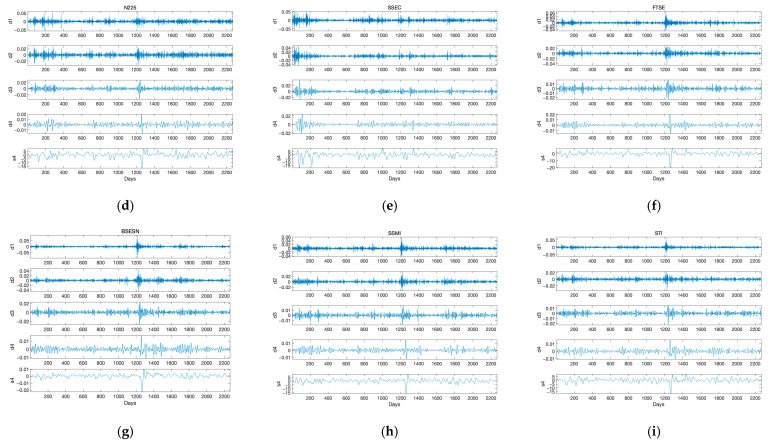
Wavelet decomposition of the 9 stock indices using MODWT. (**a**) represents the decomposed series of NYA; (**b**) represents the decomposed series of HSI; (**c**) represents the decomposed series of GDAXI; (**d**) represents the decomposed series of N225; (**e**) represents the decomposed series of SSEC; (**f**) represents the decomposed series of FTSE; (**g**) represents the decomposed series of BSESN; (**h**) represents the decomposed series of SSMI; (**i**) represents the decomposed series of STI.

To analyze multi-scale tail-risk information spillover between stock markets, we employed the wavelet decomposition method, the Maximal Overlap Discrete Wavelet Transform (MODWT). The time series decomposition was performed using the Daubechies least asymmetric (LA) filter with a length of eight (L = 8). Given that each time series comprises 2256 observations (N = 2265), and following the rule $J_{0}<\log_{2}\left(N/(L-1)+1\right)$, each time series was decomposed into five subsequences: d1, d2, d3, d4, and s4. The subsequences d1 through d4 capture high-frequency components and represent the detailed structures of the original time series, corresponding to time intervals of 2–4 days, 4–8 days, 8–16 days, and 16–32 days, respectively. Following the methodology outlined in Chen et al. [63], we categorized d1 as short-term trading (high frequency trading), d2 as short-medium term trading (high-medium frequency trading), d3 as representing mid-term trading (medium frequency trading), and d4 as long-term trading (low frequency trading). The s4 component represents the lowest frequency trend of the time series. Figure 2 demonstrates the decomposition of a time series using MODWT.

Based on the wavelet decomposition of the time series, it is evident that the volatility of the signal sequence is most pronounced at the scales d1, d2, and d3. Table 6 presents the energy contribution of fluctuations at each scale to the fluctuation of the original series. The results indicate that the energy contribution at d2 is the highest (approximately 50%), followed by d1 (nearly 25%). Therefore, short-term and short-medium term trading captures most of the energy of stock returns. Fernandez [64], Shik Lee [65], and Dajčman [66] also reached similar conclusions that stock return movements are predominantly driven by short-term fluctuations. This phenomenon can be explained by the fact that investors with shorter investment horizons tend to adjust their investment portfolios more rapidly and frequently. Since the combined energy proportions of d1, d2, and d3 account for nearly 85% of the total energy, indicating that these three scales capture the majority of return variability, we focus on the spillover effects at these three scales in the following analysis.

Table 7, Table 8 and Table 9 represent connectedness tables at scale d1, d2, and d3.

Based on the computed average effective Rényi transfer entropy, the results for d1, d2, and d3 are 0.216, 0.262, and 0.024, respectively. This indicates that the total amount of information spillover associated with extreme events is maximized in short term and short-medium term trading, particularly in short-medium term trading. Conversely, the amount of information spillover in medium-term trading drops significantly. Moreover, it can be observed that as trading frequency decreases, negative values of pairwise effective Rényi transfer entropy become increasingly prevalent, particularly in medium-term trading. This indicates that with longer trading horizons, the complexity of information spillovers becomes more pronounced. Therefore, long-term traders find it harder to hedge their risks compared to short-term traders.

To identify the roles of stock markets across different time scales, we calculate the effective Rényi transfer entropy “To” and “From” and net effective Rényi transfer entropy. At scale d1, the N225 demonstrates the highest row sum of total effective Rényi transfer entropy, while the SSEC shows the lowest value. The US stock market exhibits the largest column sum of total effective Rényi transfer entropy “To” at 0.349, compared to the Hong Kong market’s lowest value of 0.142. Negative net effective Rényi transfer entropy is found for the HSI, GDAXI, N225, FTSE, and SSMI, indicating their role as information recipients in the global network. The NYA, SSEC, BSESN, and STI indices exhibit positive net effective Rényi transfer entropy, suggesting their function as information transmitters.

At scale d2, the NYA index exhibits the highest row sum of total effective Rényi transfer entropy “From” (0.396), while the SSEC index records the lowest value (0.158). The US stock market continues to show the highest total transfer entropy “To” at 0.404, whereas the Hong Kong stock market remains the lowest at 0.163. At this scale, the N225, BSESN, and STI indices also exhibit negative net effective Rényi transfer entropy, reinforcing their status as information recipients within the network. Conversely, the NYA, HSI, GDAXI, SSEC, FTSE, and SSMI indices demonstrate positive net effective Rényi transfer entropy, indicating their role as information transmitters.

At scale d3, the BSESN index demonstrates the highest row sum of total effective Rényi transfer entropy “From” (0.087), whereas the SSEC index shows the lowest value (−0.064). The US stock market again exhibits the highest total effective Rényi transfer entropy “To” at 0.115, while the SSEC again records the lowest value (−0.045). The negative net effective Rényi transfer entropy observed for the HSI, N225, FTSE, BSESN, and STI indicates their position as information recipients in the global network. Conversely, the NYA, GDAXI, SSEC, and SSMI indices demonstrate positive net effective Rényi transfer entropy, signifying their role as information transmitters.

**Figure 3:**
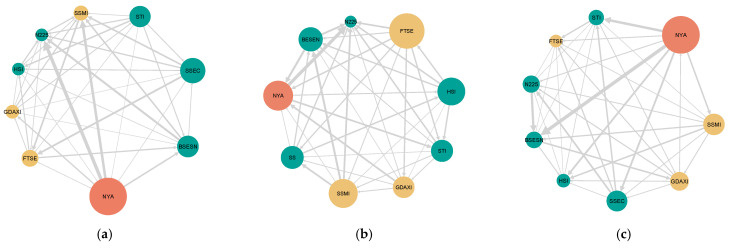
Multi-scale tail-risk information spillover network based on pairwise net effective Rényi transfer entropy. (**a**) represents the network at scale d1; (**b**) represents the network at scale d2; (**c**) represents the network at scale d3. European markets are represented by yellow nodes, Asian markets by blue nodes, and the US market by an orange node.

Figure 3 illustrates tail-risk information spillover characteristics across different trading frequencies. In each network, nodes are arranged clockwise by outdegree, with colors indicating their continental affiliation. Individual node positions vary across the three networks, showing no distinct regional patterns. This demonstrates that tail-risk information spillover displays different characteristics at varying trading frequencies without clearly discernible patterns. However, the US stock market node remains consistently large across all frequencies, and its edges to other markets are relatively thick, demonstrating its dominant influence.

Based on the results, the roles of some stock markets across different frequencies display no systematic patterns, reflecting the complexity and unpredictability of tail-risk information spillover among stock markets. However, other stock markets exhibit relatively consistent roles. First, the US stock market emerges as the largest information transmitter across all frequencies and acts as a net information exporter, demonstrating its dominant influence in the global stock market. Consequently, extreme events in the US market would substantially impact global markets. Conversely, Japan’s stock market acts as a net information receiver at all three frequencies, suggesting greater susceptibility to external information influences. A potential explanation for Japan’s role as an information receiver is its relatively slower market capitalization growth [67]. Additionally, the stock market in mainland China consistently demonstrates the lowest levels of information reception across all trading frequencies while maintaining its function as a net information transmitter, suggesting a degree of market independence. This observation aligns with the findings of Zheng and Song [68], which attributed this phenomenon to China’s stock market policies. Specifically, China’s stock market still remains insulated from global markets, limiting its capacity to attract significant inflows of external “hot money”. Furthermore, regulatory restrictions on foreign ownership of Chinese stocks have effectively curtailed participation by many large international asset management firms.

### 3.3. Robustness Test

#### 3.3.1. Robustness Test for Overall Characteristics

In the previous empirical analysis, we constructed a connectedness table using the parameter q = 0.8 to examine the overall characteristics of tail-risk information spillovers. To test the robustness of our results, we now set q = 0.7 and q = 0.9. The connectedness tables are presented in Table 10 and Table 11, respectively.

The findings are largely consistent with those obtained for q = 0.8, confirming the robustness of our conclusions. First, the US stock market consistently exhibits the highest total effective Rényi transfer entropy “To” values (0.654 for q = 0.7, 0.644 for q = 0.9), underscoring its dominant role as the largest information transmitter of tail risk. Notably, the largest spillovers from NYA are directed toward HSI (0.111 for q = 0.7, 0.236 for q = 0.9) and STI (0.094 for q = 0.7, 0.086 for q = 0.9), further confirming its significant influence in the global market. Second, the regional pattern of information spillovers remains stable. The US and European stock markets continue to function as primary information transmitters, while Asian stock markets primarily act as receivers. The only exception arises with SSEC at q = 0.9, where its net effective Rényi transfer entropy becomes slightly positive (0.002), indicating a role as an information transmitter. However, due to the small value of net effective Rényi transfer entropy, its impact on other markets is negligible.

In conclusion, the consistency of the results supports the robustness of our findings regarding tail-risk information spillovers among global stock markets.

#### 3.3.2. Robustness Test for Multi-Scale Characteristics

We also set the parameter q to 0.7 and 0.9 to analyze the characteristics of decomposed stock indices. The corresponding connectedness tables are presented in Table 11, Table 12, Table 13, Table 14, Table 15, Table 16 and Table 17.

First, the total amount of tail-risk information spillover is consistently highest during the short-to-medium trading term, and it decreases significantly as the trading term extends. Specifically, the average effective Rényi transfer entropy values for q = 0.7 are 0.251 at d1, 0.344 at d2, and 0.016 at d3; for q = 0.9, the values are 0.169 at d1, 0.247 at d2, and 0.059 at d3, respectively. These results confirm the previously observed multi-scale pattern of tail-risk information spillovers. Second, the complexity of tail-risk information spillovers increases significantly at lower trading frequencies. This is evidenced by a notable rise in the number of negative values in pairwise effective Rényi transfer entropy at scale d3 for both q = 0.7 and q = 0.9.

In terms of market roles, the positions of the US, Japanese, and Chinese stock markets remain consistent with the results for q = 0.8. The US stock market continues to be the most influential information transmitter across all frequencies, while the Japanese market remains a net information receiver. The Chinese stock market still demonstrates relative independence from global spillovers. Two minor deviations are observed: first, at scale d1 with q = 0.9, the US stock market becomes the second-largest information transmitter rather than the first; second, at scale d2 with q = 0.7, China ranks second-lowest in information reception.

Taken together, these findings reaffirm the robustness of our main conclusions based on q = 0.8, both in terms of overall characteristics and multi-scale characteristics.

## 4. Conclusions

To investigate the impact of tail risk on global stock markets, we adopt an information flow perspective. We utilize data from nine stock markets spanning from 2014 to 2024 and apply effective Rényi transfer entropy to quantify the amount of tail-risk information spillover between stock markets. In addition, we construct connectedness tables to investigate different market roles during extreme events. Furthermore, to analyze the multi-scale characteristics of information spillover, we employ the MODWT method to decompose the time series into multiple time scales. Finally, by adjusting the Rényi entropy parameter q, we verify the robustness of our empirical findings.

Our analysis uncovers key features of tail-risk information spillovers in global stock markets. First, the US and European markets serve as primary information transmitters during extreme events, with the US market showing the highest net effective Rényi transfer entropy, underscoring its important role in global tail-risk spillover. Asian markets, by contrast, act mainly as net receivers, likely due to institutional and structural differences. Second, spillover intensity peaks at short-to-medium trading horizons (4–8 days) and declines over longer periods, though spillover complexity increases. Third, while market roles vary by trading frequency, consistent patterns exist: the US consistently acts as the dominant transmitter, Japan remains a net receiver, and China exhibits limited spillover engagement, indicating relative independence.

Our findings provide valuable insights to help investors and policymakers design more effective risk management strategies for mitigating global financial risks. First, considering regional market characteristics, regulators and policymakers should clearly define their domestic markets’ roles within the global financial system to establish more robust risk regulation and defense mechanisms. When a country (region) serves as a significant information transmitter, it is essential to carry out periodic risk evaluations and take proactive measures to detect potential extreme event shocks, thereby minimizing the likelihood of such occurrences in the domestic market. Furthermore, strengthening international cooperation and establishing information-sharing mechanisms can help mitigate systemic risks. For information receivers, it is crucial to implement market-stabilizing policies, enhance information monitoring capabilities, and improve the capacity to withstand risks. Second, given that the dynamics of tail-risk information spillovers vary across different trading frequencies, investors should tailor their strategies according to their trading frequencies. High-frequency traders should monitor key market information closely and adjust positions rapidly to minimize extreme event impacts. Conversely, given the greater complexity they face, low-frequency traders should adopt more conservative strategies and strengthen risk management capacities.

In summary, this study makes several notable contributions to the literature. First, by examining tail-risk spillovers from the perspective of information entropy, our research enriches the current body of research on extreme financial risks and their spillover mechanisms. Second, we innovatively integrate effective Rényi transfer entropy with wavelet analysis to conduct a multi-scale analysis of tail-risk information spillovers. Third, we offer targeted recommendations for countries based on their roles and tailored suggestions for investors based on their trading frequencies.

However, several limitations of this research should be acknowledged. First, the analysis is confined to nine major stock markets, potentially overlooking the interconnected roles of other markets in the global market. Second, the construction of connectedness tables offers a static view of tail-risk spillovers, which may not adequately reflect the evolving nature of tail risk over time. Given these limitations, several directions for future research are worth pursuing. First, including more stock markets would provide a better understanding of global tail-risk spillover. Second, extending the analysis to other financial markets, such as cryptocurrency markets and commodity markets, would allow for investigation into cross-market tail-risk spillover. Additionally, the rolling-window approach could be applied to capture the dynamic evolution of tail risk spillovers.

## Figures and Tables

**Table 1:** **Connectedness table of stock markets.**

	$S_{1}$	$S_{2}$	…	$S_{N}$	From
$S_{1}$	$R_{11}$	$R_{12}$	…	$R_{1N}$	$\sum_{j=1}^{N}R_{1j},j\neq 1$
$S_{2}$	$R_{21}$	$R_{22}$	…	$R_{2N}$	$\sum_{j=2}^{N}R_{2j},j\neq 2$
⋮	$R_{11}$	⋮	⋮	⋮	⋮
$S_{N}$	$R_{N1}$	$R_{N2}$	…	$R_{NN}$	$\sum_{j=N}^{N}R_{Nj},j\neq N$
To	$\sum_{i=1}^{N}R_{i1},i\neq 1$	$\sum_{i=1}^{N}R_{i2},i\neq 2$	…	$\sum_{i=1}^{N}R_{iN},i\neq N$	$\frac{1}{N}\sum_{i,j=1}^{N}R_{ij},i\neq j$

**Table 2:** **Summary information of the 9 stock indices.**

Continent	Country/Region	Indices	Abbreviation
America	USA	NYSE Composite Index	NYA
Asia	Singapore	Straits Times Index	STI
	Hong Kong, China	Hang Seng Index	HSI
	China	SSE Composite Index	SSEC
	Japan	Nikkei 225	N225
	India	BSE Sensex	BSESN
Europe	Germany	Dax Index	GDAXI
	UK	FTSE 100	FTSE
	Swiss	Swiss Market Index	SSMI

**Table 3:** **Descriptive statistics.**

	Mean	Maximum	Minimum	Std. Dev.	Skewness	Kurtosis	Jarque-Bera
NYA	2.10 × 10^−4^	0.096	−0.126	0.011	−1.115	21.991	34,506.69 ***
HSI	−1.74 × 10^−4^	0.087	−0.066	0.013	0.089	6.184	959.7205 ***
GDAXI	2.13× 10^−4^	0.104	−0.131	0.012	−0.636	14.123	11,829.68 ***
N225	2.78 × 10^−4^	0.077	−0.083	0.012	−0.092	7.799	2177.066 ***
SSEC	−1.74 × 10^−4^	0.075	−0.089	0.012	−1.088	12.101	8264.668 ***
FTSE	6.99 × 10^−5^	0.087	−0.115	0.010	−0.913	16.244	16,869.68 ***
BSESN	4.35 × 10^−4^	0.086	−0.141	0.010	−1.475	26.609	53,422.92 ***
SSMI	1.08 × 10^−4^	0.068	−0.101	0.009	−0.742	12.584	8876.191 ***
STI	−9.06 × 10^−6^	0.059	−0.076	0.009	−0.431	11.698	7210.419 ***

*** *p* < 0.01.

**Table 4:** **Unconditional correlations.**

Variables	NYA	HSI	GDAXI	N225	SSEC	FTSE	BSESN	SSMI	STI
NYA	1.000								
HSI	0.259 ***	1.000							
	(0.000)								
GDAXI	0.639 ***	0.352 ***	1.000						
	(0.000)	(0.000)							
N225	0.236 ***	0.442 ***	0.326 ***	1.000					
	(0.000)	(0.000)	(0.000)						
SSEC	0.168 ***	0.540 ***	0.183 ***	0.302 ***	1.000				
	(0.000)	(0.000)	(0.000)	(0.000)					
FTSE	0.629 ***	0.378 ***	0.822 ***	0.328 ***	0.200 ***	1.000			
	(0.000)	(0.000)	(0.000)	(0.000)	(0.000)				
BSESN	0.361 ***	0.419 ***	0.450 ***	0.338 ***	0.236 ***	0.455 ***	1.000		
	(0.000)	(0.000)	(0.000)	(0.000)	(0.000)	(0.000)			
SSMI	0.564 ***	0.307 ***	0.810 ***	0.301 ***	0.178 ***	0.785 ***	0.412 ***	1.000	
	(0.000)	(0.000)	(0.000)	(0.000)	(0.000)	(0.000)	(0.000)		
STI	0.311 ***	0.563 ***	0.406 ***	0.483 ***	0.350 ***	0.430 ***	0.511 ***	0.360 ***	1.000
	(0.000)	(0.000)	(0.000)	(0.000)	(0.000)	(0.000)	(0.000)	(0.000)	

*** *p* < 0.01.

**Table 5:** **Connectedness table for the full sample.**

	NYA	HSI	GDAXI	N225	SSEC	FTSE	BSESN	SSMI	STI	From
NYA	0	0.052 ***	0.037 ***	0.031 ***	0.035 ***	0.048 ***	0.051 ***	0.029 ***	0.060 ***	0.342
HSI	0.102 ***	0	0.052 ***	0.031 ***	0.030 ***	0.049 ***	0.033 ***	0.053 ***	0.017 **	0.368
GDAXI	0.049 ***	0.011 *	0	0.032 ***	0.036 ***	0.022 **	0.032 **	0	0.025 **	0.206
N225	0.074 ***	0.007 *	0.052 ***	0	0	0.044 ***	0.040 ***	0.054 ***	0.017 *	0.288
SSEC	0.050 ***	0	0.034 **	0.029 **	0	0.032 **	0.023 *	0.049 ***	0.031 ***	0.247
FTSE	0.051 ***	0.051 ***	0	0.019 **	0.027 ***	0	0.023 **	0.019 **	0.037 ***	0.227
BSESN	0.068 ***	0.024 **	0.038 ***	0.037 ***	0.027 **	0.034 **	0	0.048 ***	0.041 ***	0.317
SSMI	0.081 ***	0.035 ***	0	0.030 ***	0.046 ***	0.013 *	0.034 ***	0	0.043 ***	0.282
STI	0.097 ***	0	0.056 ***	0.058 ***	0.040 ***	0.058 ***	0.026 **	0.068 ***	0	0.403
To	0.572	0.179	0.269	0.266	0.241	0.299	0.263	0.321	0.271	0.298
Net	0.230	−0.188	0.063	−0.023	−0.006	0.072	−0.055	0.039	−0.132	

*** *p* < 0.01, ** *p* < 0.05, * *p* < 0.1; Any off-diagonal zero values indicate statistically non-significant results (*p* > 0.1).

**Table 6:** **Scale-based energy decomposition of time series.**

	NYA	HSI	GDAXI	N225	SSEC	FTSE	BSESN	SSMI	STI
d1	20.55%	25.42%	23.37%	22.29%	25.40%	25.40%	23.93%	25.19%	22.01%
d2	55.91%	49.66%	50.50%	52.52%	48.09%	50.41%	50.75%	50.85%	50.52%
d3	11.99%	12.98%	12.82%	13.24%	13.38%	12.28%	12.46%	12.14%	12.00%
d4	5.49%	6.30%	6.69%	6.31%	7.03%	6.11%	5.92%	5.98%	7.12%
s	6.06%	5.64%	6.62%	5.64%	6.10%	5.80%	6.94%	5.84%	8.35%
Total	100%	100%	100%	100%	100%	100%	100%	100%	100%

**Table 7:** **Connectedness table at scale d1.**

	NYA	HSI	GDAXI	N225	SSEC	FTSE	BSESN	SSMI	STI	From
NYA	0	0.044 ***	0.034 ***	0.014 ***	0.003 *	0.048 ***	0.043 ***	0.015 ***	0.043 ***	0.244
HSI	0.043 ***	0	0.013 *	0.019 **	0.014 *	0.024 ***	0.013 **	0.026 ***	0.016 *	0.167
GDAXI	0.047 ***	0.016 ***	0	0.026 ***	0.008 **	0.022 ***	0.028 ***	0	0.024 ***	0.172
N225	0.061 ***	0.031 ***	0.029 ***	0	0.018 ***	0.043 ***	0.049 ***	0.036 ***	0.037 ***	0.302
SSEC	0.002 **	0.005 **	0.007 ***	0.004 **	0	0.003 ***	0.022 ***	0.038 ***	0.045 ***	0.127
FTSE	0.046 ***	0.014 ***	0.017 ***	0.047 ***	0.025 ***	0	0.032 ***	0.019 ***	0.038 ***	0.238
BSESN	0.059 ***	0.011 *	0.027 ***	0.024 ***	0.017 **	0.046 ***	0	0.021 **	0.024 ***	0.228
SSMI	0.050 ***	0.021 **	0.011 **	0.047 ***	0.057 ***	0	0.037 ***	0	0.032 **	0.255
STI	0.043 ***	0	0.031 ***	0.026 ***	0.038 ***	0.036 ***	0.015 *	0.028 **	0	0.216
To	0.349	0.142	0.169	0.207	0.178	0.222	0.240	0.183	0.259	0.216
Net	0.105	−0.026	−0.003	−0.095	0.051	−0.016	0.012	−0.072	0.043	

*** *p* < 0.01, ** *p* < 0.05, * *p* < 0.1; Any off-diagonal zero values indicate statistically non-significant results (*p* > 0.1).

**Table 8:** **Connectedness table at scale d2.**

	NYA	HSI	GDAXI	N225	SSEC	FTSE	BSESN	SSMI	STI	From
NYA	0	0.042 ***	0.064 ***	0.047 ***	0.033 ***	0.060 ***	0.063 ***	0.051 ***	0.035 ***	0.396
HSI	0.055 ***	0	0.041 ***	0.011 *	0.029 **	0.011 *	0.016 ***	0.024 **	0	0.187
GDAXI	0.037 ***	0.042 ***	0	0.061 ***	0.024 ***	0.021 ***	0.049 ***	−0.010 **	0.053 ***	0.277
N225	0.103 ***	0.033 ***	0.068 ***	0	0.049 ***	0.056 ***	0.067 ***	0.059 ***	0.040 ***	0.474
SSEC	0.029 ***	0.009 ***	0.031 ***	0.032 ***	0	0.010 **	−0.001 **	0.026 ***	0.022 ***	0.158
FTSE	0.040 ***	0.030 ***	0.001 **	0.030 ***	−0.005 *	0	0.035 ***	0.007 **	0.025 ***	0.164
BSESN	0.032 ***	0.035 ***	0.046 ***	0.038 ***	0.016 **	0.048 ***	0	0.053 ***	0.024 ***	0.290
SSMI	0.048 ***	0.027 **	0	0.031 ***	0	0.017 **	0.020 ***	0	0.027 ***	0.171
STI	0.060 ***	0.020 **	0.045 ***	0.019 **	0.018 **	0.044 ***	0.016 **	0.017 **	0	0.238
To	0.404	0.238	0.295	0.269	0.163	0.267	0.265	0.227	0.227	0.262
Net	0.009	0.050	0.019	−0.205	0.005	0.103	−0.025	0.056	−0.011	

*** *p* < 0.01, ** *p* < 0.05, * *p* < 0.1; Any off-diagonal zero values indicate statistically non-significant results (*p* > 0.1).

**Table 9:** **Connectedness table at scale d3.**

	NYA	HSI	GDAXI	N225	SSEC	FTSE	BSESN	SSMI	STI	From
NYA	0	−0.004 **	0.010 ***	−0.005 **	−0.021 *	0.014 ***	−0.001 ***	−0.018 *	0	−0.024
HSI	0.016 ***	0	0.016 ***	0	−0.002 **	0.004 **	−0.003 **	0.015 ***	−0.011 *	0.036
GDAXI	0.003 ***	0.008 ***	0	0.010 ***	−0.013 **	−0.003 **	0.024 ***	0.013 ***	0.022 ***	0.066
N225	0.003 ***	0.002 **	0.020 ***	0	0.001 ***	−0.003 ***	−0.005 ***	0.013 ***	−0.005 **	0.026
SSEC	0	−0.006 ***	−0.007 ***	−0.018 ***	0	−0.007 ***	0	−0.010 ***	−0.016 **	−0.064
FTSE	0.023 ***	0.007 ***	0.005 **	−0.004 **	0.004 **	0	0.005 ***	0.001 **	0.015 ***	0.055
BSESN	0.042 ***	−0.011 *	0.008 ***	0.022 **	0	0.007 ***	0	0.019 ***	−0.001 **	0.087
SSMI	−0.003 ***	0	0.006 ***	0.006 **	−0.015 *	−0.004 **	0.007 ***	0	−0.001 **	−0.004
STI	0.030 ***	−0.007 *	0.016 ***	−0.004 **	0	0.002 **	0.001 **	0.001 **	0	0.039
To	0.115	−0.011	0.075	0.007	−0.045	0.010	0.028	0.034	0.003	0.024
Net	0.139	−0.047	0.009	−0.019	0.019	−0.045	−0.059	0.038	−0.036	

*** *p* < 0.01, ** *p* < 0.05, * *p* < 0.1; Any off-diagonal zero values indicate statistically non-significant results (*p* > 0.1).

**Table 10:** **Connectedness table for the full sample when q = 0.7.**

	NYA	HSI	GDAXI	N225	SSEC	FTSE	BSESN	SSMI	STI	From
NYA	0	0.060 ***	0.046 ***	0.029 ***	0.040 ***	0.058 ***	0.054 ***	0.034 ***	0.076 ***	0.396
HSI	0.111 ***	0	0.058 ***	0.034 ***	0.041 ***	0.055 ***	0.042 ***	0.059 ***	0.024 **	0.423
GDAXI	0.061 ***	0.016 *	0	0.046 ***	0.046 ***	0.031 **	0.041 ***	0	0.029 **	0.269
N225	0.082 ***	0.014 **	0.059 ***	0	0.016 *	0.048 ***	0.047 ***	0.065 ***	0.022 *	0.353
SSEC	0.060 **	0	0.042 *	0.034 **	0	0.038 **	0.032 *	0.059 **	0.035 **	0.300
FTSE	0.059 ***	0.062 **	0	0.028 ***	0.033 **	0	0.032 **	0.028 **	0.047 ***	0.288
BSESN	0.079 ***	0.032 **	0.047 ***	0.046 ***	0.030 **	0.045 ***	0	0.057 ***	0.051 ***	0.386
SSMI	0.094 ***	0.043 ***	0	0.034 ***	0.050 ***	0.020 **	0.036 ***	0	0.054 ***	0.332
STI	0.108 ***	0.016 *	0.062 ***	0.067 ***	0.047 ***	0.066 ***	0.034 **	0.080 ***	0	0.479
To	0.654	0.242	0.313	0.318	0.302	0.359	0.317	0.381	0.338	0.358
Net	0.258	−0.181	0.044	−0.035	0.002	0.072	−0.068	0.049	−0.141	

*** *p* < 0.01, ** *p* < 0.05, * *p* < 0.1; Any off-diagonal zero values indicate statistically non-significant results (*p* > 0.1).

**Table 11:** **Connectedness table for the full sample when q = 0.9.**

	NYA	HSI	GDAXI	N225	SSEC	FTSE	BSESN	SSMI	STI	From
NYA	0	0.188 ***	0.035 ***	0.029 ***	0.032 ***	0.046 ***	0.046 ***	0.028 ***	0.054 ***	0.458
HSI	0.236 ***	0	0.048 ***	0.026 ***	0.022 **	0.047 ***	0.025 ***	0.050 ***	0.017 **	0.472
GDAXI	0.038 ***	0	0	0.024 **	0.030 **	0.021 **	0.025 ***	0	0.017 **	0.154
N225	0.068 ***	0	0.051 ***	0	0	0.044 ***	0.034 ***	0.053 ***	0.015 *	0.251
SSEC	0.042 ***	0	0.030 ***	0.022 **	0	0.025 **	0.020 *	0.040 ***	0.030 ***	0.209
FTSE	0.043 ***	0.040 ***	0	0.016 *	0.022 **	0	0.018 **	0.015 *	0.029 ***	0.184
BSESN	0.061 ***	0.021 **	0.035 **	0.031 ***	0.026 **	0.032 ***	0	0.038 ***	0.034 ***	0.278
SSMI	0.070 ***	0.034 ***	0	0.023 **	0.040 ***	0.015 *	0.032 ***	0	0.034 ***	0.248
STI	0.086 ***	0	0.051 ***	0.049 ***	0.032 ***	0.051 ***	0.022 **	0.061 ***	0	0.353
To	0.644	0.283	0.251	0.171	0.204	0.281	0.222	0.286	0.231	0.286
Net	0.187	−0.189	0.097	−0.079	−0.005	0.097	−0.056	0.038	−0.122	

*** *p* < 0.01, ** *p* < 0.05, * *p* < 0.1; Any off-diagonal zero values indicate statistically non-significant results (*p* > 0.1).

**Table 12:** **Connectedness table at scale d1 when q = 0.7.**

	NYA	HSI	GDAXI	N225	SSEC	FTSE	BESEN	SSMI	STI	From
NYA	0	0.057 ***	0.038 ***	0.023 ***	0.009 ***	0.053 ***	0.052 ***	0.015 ***	0.051 ***	0.298
HSI	0.053 ***	0	0.015 **	0.024 **	0.023 **	0.033 **	0.018 **	0.030 ***	0.021 **	0.216
GDAXI	0.053 ***	0.023 ***	0	0.038 ***	0.014 ***	0.026 ***	0.033 ***	−0.017 *	0.036 ***	0.206
N225	0.074 ***	0.035 ***	0.033 ***	0	0.024 ***	0.048 ***	0.058 ***	0.043 ***	0.046 ***	0.361
SSEC	0.004 ***	0.003 **	0.011 ***	0.013 ***	0	0.015 ***	0.003 ***	0.037 ***	0.027 ***	0.113
FTSE	0.057 ***	0.024 ***	0.020 ***	0.063 ***	0	0	0.042 ***	0.007 ***	0.032 ***	0.245
BESEN	0.069 ***	0.014 **	0.032 ***	0.034 ***	0.023 ***	0.054 ***	0	0.058 ***	0.037 ***	0.321
SSMI	0.057 ***	0.029 ***	0.008 **	0.055 ***	0.015 **	0.024 ***	0.024 ***	0	0.037 ***	0.248
STI	0.054 ***	0.010 *	0.041 ***	0.036 ***	0.021 **	0.053 ***	0.022 **	0.017 **	0	0.253
To	0.420	0.196	0.197	0.285	0.129	0.305	0.253	0.188	0.288	0.251
Net	0.122	−0.020	−0.009	−0.076	0.016	0.060	−0.068	−0.060	0.035	

*** *p* < 0.01, ** *p* < 0.05, * *p* < 0.1; Any off-diagonal zero values indicate statistically non-significant results (*p* > 0.1).

**Table 13:** **Connectedness table at scale d2 when q = 0.7.**

	NYA	HSI	GDAXI	N225	SSEC	FTSE	BSESN	SSMI	STI	From
NYA	0	0.055 ***	0.073 ***	0.066 ***	0.041 ***	0.073 ***	0.084 ***	0.069 ***	0.040 ***	0.501
HSI	0.064 ***	0	0.043 ***	0.008 **	0.032 **	0	0.041 ***	0.028 ***	0	0.216
GDAXI	0.053 ***	0.047 ***	0	0.073 ***	0.030 ***	0.021 ***	0.060 ***	−0.012 ***	0.064 ***	0.335
N225	0.123 ***	0.044 ***	0.083 ***	0	0.059 ***	0.063 ***	0.080 ***	0.076 ***	0.049 ***	0.577
SSEC	0.042 ***	0.018 ***	0.038 ***	0.039 ***	0	0.004 ***	0.032 ***	0.049 ***	0.061 ***	0.284
FTSE	0.058 ***	0.039 ***	0.013 ***	0.035 ***	0.027 ***	0	0.042 ***	0.027 ***	0.049 ***	0.289
BSESN	0.036 ***	0.043 ***	0.054 ***	0.048 ***	0.022 **	0.055 ***	0	0.025 ***	0.027 ***	0.310
SSMI	0.058 ***	0.033 **	0	0.042 ***	0.067 ***	0	0.046 **	0	0.038 ***	0.285
STI	0.072 ***	0.022 **	0.050 ***	0.019 ***	0.047 ***	0.043 ***	0.019 *	0.028 ***	0	0.300
To	0.506	0.302	0.354	0.330	0.323	0.259	0.403	0.289	0.329	0.344
Net	0.005	0.086	0.020	−0.247	0.039	−0.031	0.094	0.004	0.029	

*** *p* < 0.01, ** *p* < 0.05, * *p* < 0.1; Any off-diagonal zero values indicate statistically non-significant results (*p* > 0.1).

**Table 14:** **Connectedness table at scale d3 when q = 0.7.**

	NYA	HSI	GDAXI	N225	SSEC	FTSE	BSESN	SSMI	STI	From
NYA	0	−0.008 **	0.014 ***	−0.003 ***	−0.018 **	0.022 ***	−0.003 ***	−0.023 **	0	−0.020
HSI	0.013 ***	0	0.014 ***	0	0.003 ***	−0.004 ***	−0.002 ***	0.016 ***	−0.014 *	0.025
GDAXI	−0.003 ***	0.014 ***	0	0.014 ***	−0.012 **	−0.010 ***	0.034 ***	0.012 ***	0.025 ***	0.073
N225	−0.004 **	0.008 ***	0.016 ***	0	0.007 ***	−0.002 **	−0.005 ***	0.013 ***	−0.001 ***	0.031
SSEC	−0.038 *	−0.004 ***	−0.006 ***	−0.015 **	0	−0.008 ***	−0.030 **	−0.012 ***	−0.017 ***	−0.130
FTSE	0.018 ***	0.007 ***	0.002 **	−0.004 **	0.003 ***	0	0.007 ***	−0.005 **	0.015 ***	0.042
BSESN	0.046 ***	−0.009 **	0.005 **	0.028 ***	−0.008 **	0.013 ***	0	0.021 ***	0.001 **	0.096
SSMI	−0.012 **	0.006 ***	0.004 ***	0.004 ***	−0.018 **	−0.010 **	0.014 ***	0	−0.003 ***	−0.015
STI	0.028 ***	−0.005 **	0.016 ***	−0.002 **	0	−0.002 **	0.003 ***	0.004 **	0	0.041
To	0.046	0.009	0.064	0.022	−0.043	−0.001	0.018	0.025	0.005	0.016
Net	0.065	−0.016	−0.009	−0.010	0.086	−0.043	−0.078	0.040	−0.036	

*** *p* < 0.01, ** *p* < 0.05, * *p* < 0.1; Any off-diagonal zero values indicate statistically non-significant results (*p* > 0.1).

**Table 15:** **Connectedness table at scale d1 when q = 0.9.**

	NYA	HSI	GDAXI	N225	SSEC	FTSE	BESEN	SSMI	STI	From
NYA	0	0.034 ***	0.035 ***	0.012 **	0	0.047 ***	0.040 ***	0.022 ***	0.035 ***	0.225
HSI	0.039 ***	0	0.012 *	0.018 *	0	0.017 **	0.013 **	0.023 **	0.012 *	0.133
GDAXI	0.040 ***	0.010 *	0	0.026 ***	0.008 *	0.020 ***	0.028 ***	0.004 *	0.021 **	0.157
N225	0.050 ***	0.024 ***	0.028 ***	0	0.016 **	0.042 ***	0.039 ***	0.035 ***	0.027 ***	0.260
SSEC	0.001 *	0	0.004 *	0	0	0.005 *	0.003 *	0.020 **	0.017 **	0.050
FTSE	0.035 ***	0.010 **	0.021 ***	0.035 ***	0	0	0.030 ***	0.012 **	0.016 **	0.159
BESEN	0.049 ***	0	0.028 ***	0.017 **	0	0.042 ***	0	0.040 ***	0.021 **	0.197
SSMI	0.046 ***	0.016 **	0.016 **	0.038 ***	0	0.017 **	0.017 **	0	0.017 **	0.165
STI	0.033 ***	0	0.027 **	0.021 **	0.021 **	0.039 ***	0.015 *	0.024 **	0	0.180
To	0.292	0.094	0.170	0.166	0.044	0.229	0.185	0.181	0.166	0.169
Net	0.067	−0.039	0.013	−0.094	−0.006	0.070	−0.012	0.015	−0.014	

*** *p* < 0.01, ** *p* < 0.05, * *p* < 0.1; Any off-diagonal zero values indicate statistically non-significant results (*p* > 0.1).

**Table 16:** **Connectedness table at scale d2 when q = 0.9.**

	NYA	HSI	GDAXI	N225	SSEC	FTSE	BSESN	SSMI	STI	From
NYA	0	0.038 ***	0.064 ***	0.035 ***	0.026 ***	0.056 ***	0.050 ***	0.043 ***	0.027 ***	0.338
HSI	0.051 ***	0	0.038 ***	0.015 *	0.029 **	0.020 ***	0.028 **	0.028 ***	0	0.208
GDAXI	0.032 ***	0.038 ***	0	0.049 ***	0.024 ***	0.024 ***	0.042 ***	0	0.045 ***	0.254
N225	0.080 ***	0.023 ***	0.054 ***	0	0.044 ***	0.050 ***	0.051 ***	0.049 ***	0.027 ***	0.376
SSEC	0.025 ***	0.006 **	0.025 ***	0.023 ***	0	0.002 **	0.016 *	0.029 ***	0.036 ***	0.161
FTSE	0.034 ***	0.023 ***	0.002 *	0.025 ***	0.026 *	0	0.031 ***	0.013 ***	0.029 ***	0.182
BSESN	0.034 ***	0.026 ***	0.042 ***	0.032 ***	0.017 ***	0.040 ***	0	0.022 **	0.017 **	0.230
SSMI	0.044 ***	0.025 **	0	0.031 ***	0.047 ***	0.016 *	0.034 ***	0	0.027 **	0.224
STI	0.053 ***	0.017 **	0.040 ***	0.021 **	0.034 ***	0.037 ***	0.016 *	0.034 ***	0	0.252
To	0.352	0.196	0.264	0.230	0.247	0.244	0.267	0.217	0.209	0.247
Net	0.014	−0.012	0.010	−0.147	0.086	0.062	0.037	−0.007	−0.043	

*** *p* < 0.01, ** *p* < 0.05, * *p* < 0.1; Any off-diagonal zero values indicate statistically non-significant results (*p* > 0.1).

**Table 17:** **Connectedness table at scale d3 when q = 0.9.**

	NYA	HSI	GDAXI	N225	SSEC	FTSE	BSESN	SSMI	STI	From
NYA	0	0.004 **	0.011 ***	−0.006 *	0	0.011 ***	0.003 **	−0.010 *	0.002 **	0.015
HSI	0.027 ***	0	0.019 ***	−0.006 *	−0.001 **	0.009 ***	0	0.019 ***	0.002 ***	0.069
GDAXI	0.017 ***	0.009 **	0	0.010 ***	−0.006 *	0.009 ***	0.022 ***	0.018 ***	0.017 ***	0.095
N225	0.018 ***	0.002 **	0.025 ***	0	0	0.004 **	−0.005 **	0.019 ***	−0.001 ***	0.063
SSEC	0	−0.005 **	−0.001 **	0	0	−0.005 **	0	0	0	−0.011
FTSE	0.027 ***	0.007 **	0.007 **	0.002 *	0.008 **	0	0.009 **	0.007 **	0.017 ***	0.083
BSESN	0.040 ***	0	0.012 ***	0.015 ***	0	0.011 **	0	0.023 ***	0.003 **	0.103
SSMI	0.013 ***	0.002 **	0.012 ***	0.012 ***	−0.003 **	0.005 **	0.008 ***	0	0.005 **	0.053
STI	0.032 ***	−0.001 *	0.018 ***	0	0	0.006 **	0.001 **	0.008 **	0	0.063
To	0.173	0.016	0.102	0.026	−0.002	0.048	0.037	0.085	0.046	0.059
Net	0.158	−0.053	0.008	−0.037	0.009	−0.035	−0.065	0.032	−0.018	

*** *p* < 0.01, ** *p* < 0.05, * *p* < 0.1; Any off-diagonal zero values indicate statistically non-significant results (*p* > 0.1).

## Data Availability

The original data presented in the study are openly available in Renyi-Entropy-Dataset at https://github.com/JJJ-0812/Renyi-Entropy-Dataset (accessed on 27 March 2025).
